# BMSCs-Seeded Interpenetrating Network GelMA/SF Composite Hydrogel for Articular Cartilage Repair

**DOI:** 10.3390/jfb14010039

**Published:** 2023-01-10

**Authors:** Kaiwen Zheng, Xu Zheng, Mingzhao Yu, Yu He, Di Wu

**Affiliations:** 1 Department of Orthopaedic Surgery, Shanghai Sixth People’s Hospital, No.600 Yishan Road, Shanghai 200233, China; 2 Department of Plastic Surgery, Plastic Surgery Hospital, Peking Union Medical College and Chinese Academy of Medical Sciences, Beijing 100144, China

**Keywords:** hydrogel, interpenetrated network, articular cartilage, osteochondral defect

## Abstract

Because of limited self-healing ability, the treatment of articular cartilage defects is still an important clinical challenge. Hydrogel-based biomaterials have broad application prospects in articular cartilage repair. In this study, gelatin methacrylate (GelMA)and silk fibroin (SF) were combined to form a composite hydrogel with an interpenetrating network (IPN) structure under ultraviolet irradiation and ethanol treatment. Introducing silk fibroin into GelMA hydrogel significantly increased mechanical strength as compressive modulus reached 300 kPa in a GelMA/SF-5 (50 mg/mL silk fibroin) group. Moreover, composite IPN hydrogels demonstrated reduced swelling ratios and favorable biocompatibility and supported chondrogenesis of bone mesenchymal stem cells (BMSCs) at day 7 and day 14. Additionally, significantly higher gene expressions of *Col-2*, *Acan*, and *Sox-9* (*p* < 0.01) were found in IPN hydrogel groups when compared with the GelMA group. An in vivo study was performed to confirm that the GelMA-SF IPN hydrogel could promote cartilage regeneration. The results showed partial regeneration of cartilage in groups treated with hydrogels only and satisfactory cartilage repair in groups of cell-seeded hydrogels, indicating the necessity of additional seeding cells in hydro-gel-based cartilage treatment. Therefore, our results suggest that the GelMA/SF IPN hydrogels may be a potential functional material in cartilage repair and regeneration.

## 1. Introduction

Due to the avascular and low cellularity nature, cartilage has a limited intrinsic healing capacity when injured. When cartilage defects are left untreated, the joint deteriorates over time due to ongoing mechanical degeneration and loss of cartilage tissue, leading to osteoarthritis, the most common cause of disability [1,2]. Current clinical treatment strategies for osteochondral defects include bone marrow stimulation, autologous chondrocyte implantation, and osteochondral autografts and allografts [1,3]. Despite their widespread clinical use, these techniques are not long-term clinical solutions, prompting the development of regenerative medicine and tissue engineering approaches [4].

Hydrogels are crosslinked hydrophilic polymer networks that can swell in water to capture many times their original mass [5]. Combining comonomers, crosslink density and varying synthetic conditions can alter their physicochemical properties. Therefore, they are widely used in bio-medical fields, including tissue engineering and regenerative medicine [6]. Gelatin methacrylate (GelMA) is a gelatin-derived, photopolymerizable biomaterial with good cell adhesion, low immunogenicity, and favorable formability [7]. GelMA is produced by reacting gelatin with methacrylic anhydride (MA). Due to the methacryloyl groups, when a photo-crosslink initiator is added, GelMA undergoes photoinitiated radical polymerization to form covalently crosslinked hydrogels. Thus, GelMA has been used to reconstruct osteochondral lesions in the knee joint as it provides a scaffolding matrix for cell migration and new tissue generation [5]; however, mechanical strength is the critical property of hydrogels used in cartilage tissue repair. Although the mechanical strength of GelMA hydrogels is tunable, it is still unable to support cartilage regeneration [6].

Silk fibroin (SF), extracted from silkworm silk, is a unique natural protein used for tissue engineering due to many desired physiochemical properties, such as excellent cytocompatibility, unique mechanical property, non-toxic degradation products, controllable biodegradability, and desirable processability [8]. Compared to other polymers, an advantage of SF is its ability to crosslink without any chemical modifications [9]. The cross-linking process is related to the conformational change from a random coil to a β-sheet [10]. Moreover, SF scaffolds can support chondrocyte maturation and significantly enhance the production of cartilage matrix deposition [11,12]. Previously, it has been reported that SF and GelMA could form an interpenetrating polymer network (IPN) with notably enhanced mechanical strength and biocompatibility. However, there is lower enzymatic degradation compared to GelMA hydrogel [9,13]. Meanwhile, the GelMA/SF composite hydrogel has the unique advantage of high adhesion to the native tissue, which would avoid shedding implants and promote early-term integration and cell migration [14].

**Figure 1:**
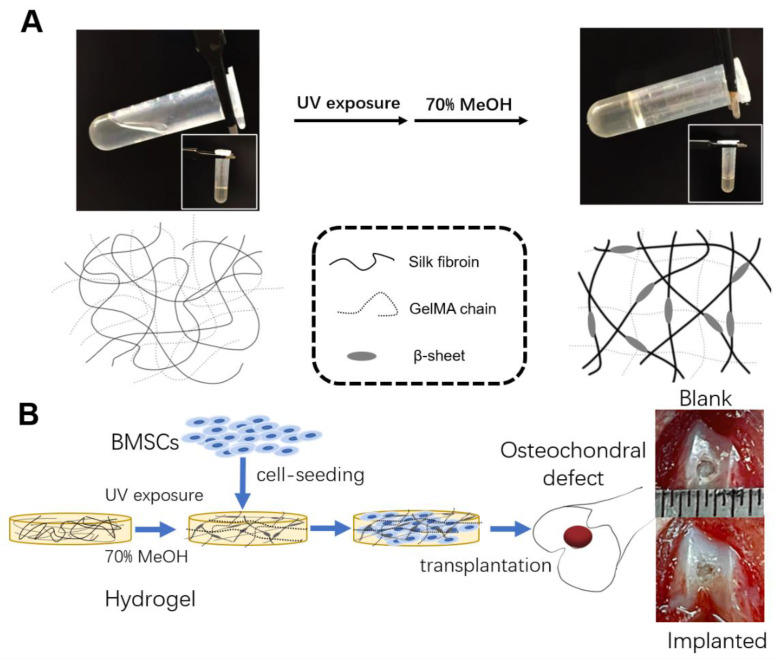
(**A**) Schematic for synthesizing GelMA/SF composite IPN hydrogels: to create the GelMA–SF IPN hydrogel, the GelMA was mixed with SF solution and crosslinked under UV exposure in the presence of a photoinitiator, following treatment with 70% methanol to induce SF crystallization and (**B**) in vivo articular cartilage repair study.

In this study, we added SF to notably enhance the strength of a pure GelMA hydrogel to fabricate an IPN composite hydrogel (Figure 1A). Its physical and biochemical properties were evaluated in vitro. Furthermore, this GelMA/SF composite hydrogel, with or without bone mesenchymal stem cells (BMSCs), was implanted into a rat knee osteochondral defect site to investigate its feasibility in vivo (Figure 1B). We hypothesized that this composite hydrogel would demonstrate outstanding potential for use in articular cartilage repair or cartilage tissue engineering.

## 2. Materials and Methods

All animal experiments in this study were carried out following the Animal Ethics Committee of Peking Union Medical College Hospital (No.XHDW-2021-093) and strictly adhered to The Guide for the Care and Use of Laboratory Animals (GB14925-2010; NIH).

### 2.1. Preparation of GelMA

GelMA was synthesized according to previous protocols [5,9]. Briefly, 10 wt% gelatin (derived from porcine skin, Sigma-Aldrich, St Louis, MO, USA) was dissolved in PBS at 50 °C and added with 5 mL of methacrylic anhydride (MA) at a rate of 0.5 mL/min under continuous stirring for 2 h. The GelMA solution was diluted with four times the volume of PBS and dialyzed against distilled water using a 12–14 kDa cutoff dialysis tube at 50 °C for 6 days. The GelMA solution was frozen at −80 °C, lyophilized, and stored at −80 °C until further use.

### 2.2. Synthesis of GelMA/SF Composite Hydrogels

GelMA/SF composite hydrogels were synthesized following the previous protocol [9]. Briefly, the GelMA solution was mixed with different SF concentrations (Sigma-Aldrich, St Louis, MO, USA) to produce different hydrogel formulas. The composite hydrogels were named for different final SF concentrations, as shown in Table 1. The photoinitiator Irgacure 2959 (Sigma-Aldrich, St Louis, MO, USA) was added to make a final concentration of 1 wt%. The premixed solution was continuously mixed at room temperature for 2 h to obtain homogenous mixtures. When transferred to different molds, the solution was exposed to UV light (320–500 nm, 7.0 mW·cm^−2^) for 2 min to photo-crosslink. Then, the hydrogel was immersed in 70% ethanol solution at room temperature for 1 h, removed, and rinsed with PBS solution thoroughly.

### 2.3. Characterization of GelMA/SF Composite Hydrogels

#### 2.3.1. Structural Analysis

Fourier Transform infrared spectroscopy (FTIR) analysis was conducted to perform structural analysis. The FTIR analysis was conducted on a Fourier transform infrared spectrometer (FTIR-7600, Lambda Scientific Systems, Miami, FL, USA). The scan was performed from 600 to 4000 cm^−1^. Synthesized hydrogels of different formulas were lyophilized before tests.

#### 2.3.2. Scanning Electron Microscopy Analysis

To perform electron microscopy for the GelMA/SF composite hydrogels, the swollen hydrogel samples were cut into small bulks, frozen in liquid nitrogen, lyophilized, and coated with gold. The samples were observed using a scanning electron microscope (High-Tech SU8010, Hitachi, Tokyo, Japan). Captured images were analyzed using the software ImageJ to determine the porosity. At least five samples from every group were used for the quantification.

### 2.4. Physical Properties of GelMA/SF Composite Hydrogels

#### 2.4.1. Mechanical Testing

The mechanical properties of the GelMA/SF composite hydrogels were characterized by compressive stress–strain measurements on Universal Testing Systems (Instron 5569, Instron, Norwood, MA, USA) at room temperature. The crosslinked GelMA/SF composite hydrogels were immersed in PBS solution and then compressed at a rate of 0.3 mm/min until failure. The compressive modulus was determined as the slope of the linear region of the stress–strain curve in the 0–10% strain range. Each experiment contains five replicates.

#### 2.4.2. Swelling Ratio Measurement

To assess the swelling ratios, the crosslinked GelMA/SF composite hydrogels were immersed in PBS solution at room temperature for 24 h. Excess surface water was removed with filter paper and each hydrogel was weighed and recorded as wet weight. The samples were then lyophilized, weighed, and recorded as dry weight. The swelling ratios were calculated, as shown in Equation (1). Each experiment contains four replicates.

(1) $$\mathrm{swelling}\;\mathrm{ratio}=\frac{\mathrm{wet}\;\mathrm{weight}-\mathrm{dry}\;\mathrm{weight}}{\mathrm{dry}\;\mathrm{weight}}$$

#### 2.4.3. Degradation Test

To perform the degradation test, the lyophilized samples of crosslinked GelMA/SF composite hydrogels were immersed in PBS solution containing 2 U/mL collagenase type II at 37 °C. After 1, 3, 5, and 7 days, samples were removed, lyophilized, and weighed. The degradation ratio was calculated as the dry weight ratio at each time point to the original dry weight Equation (2). Each experiment contains four replicates.

(2) $$\mathrm{degradation}\;\mathrm{ratio}\left(\%\right)=\frac{\;\mathrm{original}\;\mathrm{dry}\;\mathrm{weight}-\mathrm{dry}\;\mathrm{weight}\;\mathrm{after}\;\mathrm{degradation}}{\mathrm{original}\;\mathrm{dry}\;\mathrm{weight}}\times 100$$

### 2.5. Cell Viability, Proliferation, and Differentiation on Composite Hydrogels

Rat BMSC cells were purchased from the Chinese Academy of Sciences Cell Bank (Serial: SCSP-402). BMSCs were cultured in α-MEM medium supplemented with 10% FBS and 1% Penicillin-Streptomycin under 37 °C and 5% CO_2_ and were passaged at 80% confluence. The culture medium was changed three times a week. BMSCs in passage 5 were studied. Before cell seeding, the GelMA/SF hydrogel solution was treated with UV exposure and 70% ethanol and then rinsed twice with a sterilized PBS solution. Then, BMSCs were harvested after cell digestion with 2% trypsin and resuspended in the cell culture medium. Suspensions were adjusted at a density of 1 × 10^5^ cells/mL before seeding onto the surface of the hydrogel in the respective containers. Each experiment has three replicates. The method was the same for the BMSC-seeded hydrogel preparation in vivo study.

CCK-8 assays determined cell proliferation in 96-well plates. After 1, 3, and 5 days of cell seeding, the culture medium of each well was replaced with 100 μL fresh culture medium supplemented with 10 μL CCK-8 reagent. Samples were incubated for 2 h at 37 °C. The cell proliferation was presented as the optical density detected at 450 nm with a microplate reader (Varioskan™ LUX, Thermo Fisher Scientific, Waltham, MA, USA).

Cell viability and differentiation were performed in 24-well plates. Cell viability was evaluated with Calcein AM/PI Double Stain Kit (FS1161-500T, Shanghai Fushen Biotechnology, Shanghai, China) following the manufacturer’s instructions. After 6, 12, 24, and 48 h after seeding, the culture medium was removed and samples were rinsed with sterilized with PBS solution. Then, 1 mL of PBS containing 4.5 μM PI and 4 μM Calcein-AM was added to each well. Then, they were incubated for 30 min at 37 °C protected from light, and visualized under a fluorescence microscope. To induce chondrogenic differentiation, BMSCs were cultured in 1 mL of chondrogenic medium (RAXMX-90041, Cyagen Biosciences, Guangzhou, China). The chondrogenic medium was changed twice a week.

### 2.6. qPCR Analysis

After 7 and 14 days of chondrogenic differentiation, total RNA was isolated and complementary DNA (cDNA) was prepared using the RNA purification kit and 4 × EZscript Reverse Transcription Mix II kit (EZBioscience, Roseville, MN, USA) according to the manufacturer’s instructions. Real-time quantitative PCR was performed using the SYBR Green Master Mix (EZBioscience, Roseville, MN, USA) in a real-time PCR System (QuantStudio™ 7 Flex, Thermo Fisher Scientific, Waltham, MA, USA). The reaction was performed with a total volume of 10 μL in each reaction well under the following conditions: 95 °C for 5 min to activate, followed by 40 cycles of 10 s at 95 °C and 60 s at 60 °C. The quantitative gene expression was normalized to glyceraldehyde 3-phosphate dehydrogenase (GAPDH), and the relative expression was determined using the ΔΔCt method. Each sample has three technical replicates. Primer sequences are listed in Table 2.

### 2.7. In Vivo Rat Osteochondral Defect Repair Models

Although higher SF concentrations contribute to higher mechanical properties, they will form tighter structures and inhibit cell proliferation according to the results of SEM and CCK-8 tests in vitro. Taking these aspects into account, GelMA/SF-3 (GelMA: 60 mg/mL and SF: 30 mg/mL) was chosen for further in vivo study. The preparation of GelMA/SF IPN hydrogels and BMSCs-seeded hydrogels is described in the methods part.

Male SD rats that were 10 weeks old were randomly divided into three groups (6 knees per group). Different groups were defined as follows: Blank group (defect only, untreated), Gel only group (defect filled with GelMA/SF hydrogel), and Gel + Cells group (defect filled with BMSC-loaded GelMA/SF hydrogel). After general anesthesia and sterilizing, the knee joints were opened and the patella was dislocated. A drill was used to make osteochondral defects (diameter = 1.5 mm, depth = 1 mm) on the center of the trochlear groove. The joint capsule and skin were then closed. The rats were allowed to move freely in the cage after the operation.

### 2.8. Histological and Immunohistochemical Analysis

The rats were euthanized at 6 and 12 weeks post-surgery. The defect sites on femur grooves were imaged for quantitative evaluation by the International Cartilage Repair Society (ICRS) macroscopic score [15]. All samples from each group were fixed in 4% paraformaldehyde, decalcified in 10% EDTA, embedded in paraffin, cut into 6 μm slices, and mounted onto adhesive slides. Sections were stained with Hematoxylin-Eosin (H-E) and Safranin-O staining and evaluated with the ICRS Visual Histological Assessment Scale (ICRS-VHAS) and the O’Driscoll score [16,17]. Collagen type I and II production was examined through immunohistochemical staining. Briefly, after deparaffinization, rehydration, and antigen retrieval using Tris-EDTA, sections were incubated with rabbit antibodies against collagen type II (1:100, Affinity Biosciences, Cincinnati, OH, USA), followed by HRP-conjugated goat anti-rabbit secondary antibody (1:200, Affinity Biosciences, Cincinnati, OH, USA). The immunocomplex area was visualized by chromogen 3,3′-diaminobenzidine (DAB, Beyotime Biotechnology, Haimen, China) for 5 min.

### 2.9. Statistical Analysis

Two blinded observers evaluated all histological scores. The data are presented as the mean ± standard deviation. One-way analysis of variance (ANOVA) followed by Tukey’s multiple comparison tests was used to determine the significant differences using Prism 8.0 software (GraphPad). A value of *p* < 0.05 was considered to indicate a significant difference.

## 3. Results

### 3.1. Characterization of GelMA/SF Composite Hydrogels Formation

This study aimed to fabricate an IPN composite hydrogel composed of GelMA and SF. Briefly, 10 wt% gelatin was with methacrylic anhydride (MA) under continuous stirring. The GelMA solution was diluted and dialyzed against distilled water. The GelMA solution was mixed with different SF concentrations to produce different hydrogel formulas. The composite hydrogels were named for different final SF concentrations, as shown in Table 1. The photoinitiator Irgacure 2959 was added to make a final concentration of 1 wt%. The premixed solution was continuously mixed, exposed to UV light, immersed in 70% ethanol solution, and rinsed with PBS solution thoroughly before further use.

#### 3.1.1. Structural Analysis

**Figure 2:**
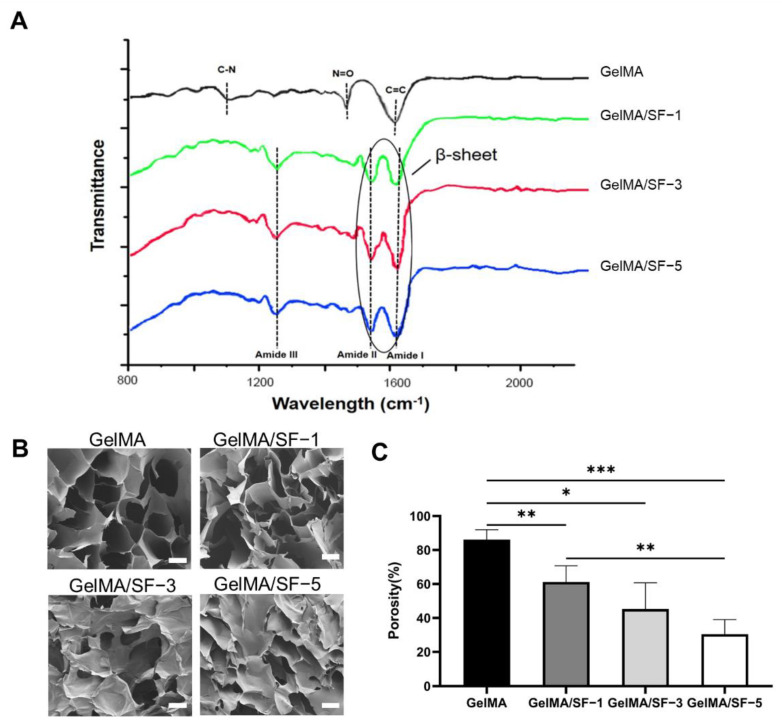
Characterization of GelMA/SF composite hydrogels formation. (**A**) Structural characterization of the hydrogels was performed by FTIR analysis indicating that the β-sheet structure was formed in all the hydrogel groups containing SF. (**B**) Representative SEM images of different hydrogels (scale bar = 50 μm). With a higher concentration of SF, porous structures changed into lamellar. (**C**) The porosity of each hydrogel was determined based on the SEM images (n = 5). Scale bar = 50 μm; significant differences symbols: * = *p* < 0.05; ** = *p* < 0.01; *** = *p* < 0.001.

Structural characterization of the GelMA/SF composite hydrogels was performed by FTIR analysis. As shown in Figure 2A, the typical amide-I (C=O stretching), amide-II (N–H bending), and amide-III (C-N absorbance) bands for the different composite hydrogel groups were all presented in the specific region of the β-sheet structure (1620 cm^−1^, 1531 cm^−1^, and 1230 cm^−1^ respectively). These bands indicate the transition into the β-sheet structure in all the composite hydrogel groups, not pure GelMA hydrogel.

#### 3.1.2. Microstructure Characterization

Since it is essential to facilitate the diffusion and exchange of nutrients and metabolic waste of seeded cells by controlling the internal microstructure and porosity, cross-sectional microstructures of hydrogels were examined by SEM. As is shown in Figure 2B, GelMA hydrogels showed a typical highly porous structure. As the SF concentration increased, the composite hydrogels’ microstructures changed from highly porous to semi-lamellar structures, as previously reported, as the typical microstructure of the SF hydrogel [18]. Moreover, SF and GelMA hydrogels had good compatibility with each other, as there were no obvious phase separation phenomena in any of the GelMA/SF IPN hydrogels, similar to a previous study [9]. Porosity analysis based on the captured SEM captured also confirmed the findings (Figure 2C).

### 3.2. Physical Properties of GelMA/SF Composite Hydrogels

#### 3.2.1. Mechanical Properties

**Figure 3:**
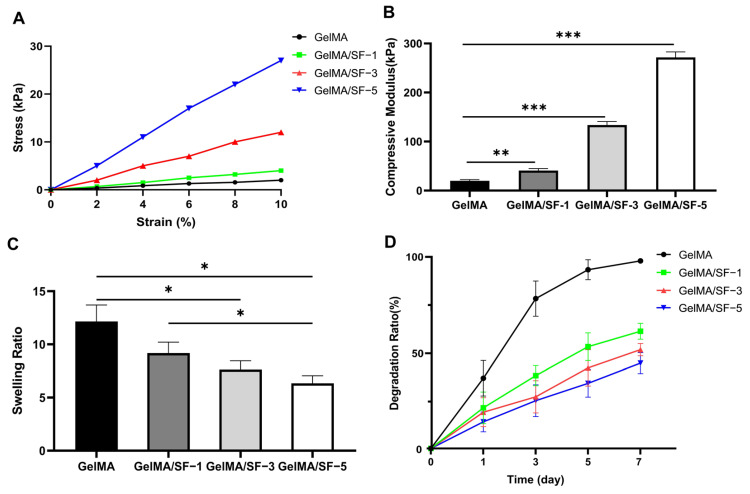
Physical properties of GelMA/SF composite hydrogels. (**A**) The representative stress-strain curves of hydrogels with different formations. (**B**) The compressive modulus of each hydrogel (n = 5). The formation of the IPN structure significantly increased the compressive modulus of the GelMA/SF composite hydrogels. (**C**) The swelling property of each hydrogel in PBS solution at room temperature for 24 h (n = 4). GelMA/SF composite hydrogels showed significantly reduced degradation rates compared to GelMA alone. (**D**) Degradation ratio of each hydrogel in PBS solution containing 2 U/mL collagenase type II at 37 °C after 1, 3, 5, and 7 days (n = 4). GelMA/SF composite hydrogels displayed a significant reduction in the degradation ratios compared to GelMA groups (*p* < 0.05). Furthermore, higher SF concentration contributed to lower degradation rates. Significant differences symbols: * = *p* < 0.05; ** = *p* < 0.01; *** = *p* < 0.001.

The mechanical properties of GelMA/SF composite hydrogels were determined using an unconfined, uniaxial compression test. The compressive modulus was calculated from the slope of the stress-strain curve’s 0–10% strain (Figure 3A). The statistical analysis illustrated a significant effect of SF concentration on the compressive modulus of the composite hydrogels (Figure 3B) because the SF hydrogel is significantly stiffer than the GelMA hydrogel. As the GelMA concentration further increased, the compressive modulus of the hydrogel therewith decreased with statistical differences (*p* < 0.01).

#### 3.2.2. Swelling Ratio

Swelling ratios of different hydrogels were calculated as the ratio of the weight difference between fully hydrated and dried hydrogels to fully hydrated hydrogels, as shown in Figure 3B. Pure GelMA hydrogel had the highest swelling ratio, which was significantly higher than GelMA/SF-3 and GelMA/SF-5 groups. When SF was added to the hydrogels, the swelling ratios decreased. The hydrogel of group GelMA/SF-5 had the lowest swelling ratio, indicating that higher SF concentrations could lead to a lower swelling ratio.

#### 3.2.3. Degradation of GelMA/SF Composite Hydrogels

Manipulating the degradation of hydrogels to fit the tissue repair rate is important for the rational design of hydrogel-based biomaterials for cartilage and osteochondral tissue regeneration [1]. The enzyme-mediated degradation properties of hydrogels under collagenase II were investigated in vitro (Figure 3C). The results showed that all hydrogels underwent rapid degradation in the collagenase solution. Pure GelMA hydrogel had the highest degradation ratio at every time point. In addition, GelMA/SF composite hydrogels significantly reduced the degradation ratio, depending on their GelMA concentration. The SF component in the hydrogel could greatly reduce the rate and extent of enzymatic degradation. The possible explanation for these results is that, at higher SF concentrations, more β-sheet structures provide a more compact structure of composite hydrogels, hindering the permeation of collagenase solution into the hydrogel [19].

### 3.3. Cell Viability, Proliferation, and Chondrogenesis in Hydrogels

**Figure 4:**
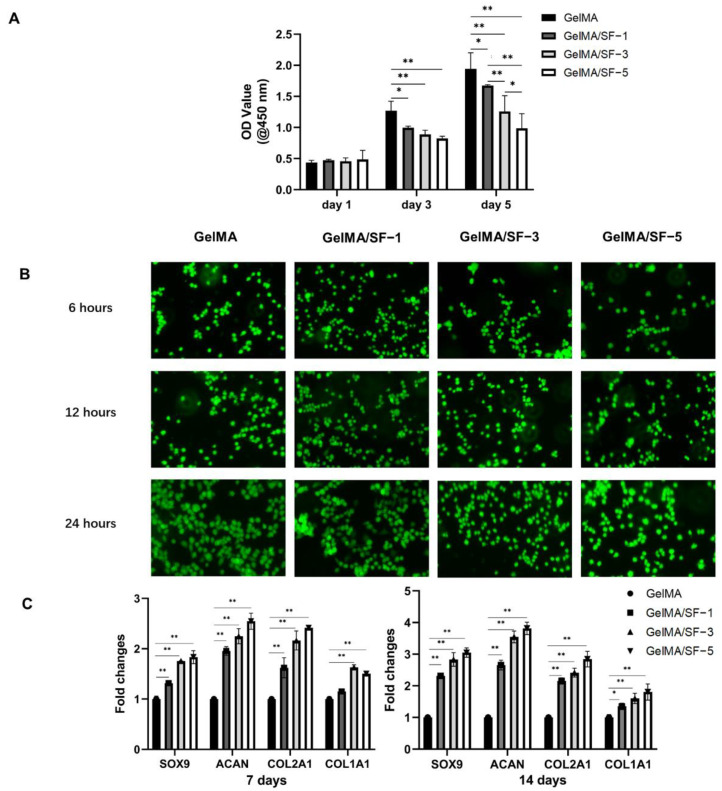
Cell viability, proliferation, and chondrogenesis in hydrogels. (**A**) Cell viability of rat BMSCs seeded on different hydrogels at different time points, presented as OD at 450 nm via a microplate reader (n = 5). (**B**) The representative fluorescent images of rat BMSCs seeded on different hydrogels at different time points with Calcein-AM/PI double staining (Magnification: 200×). (**C**) Cartilage-related gene expression changes of rat BMSCs loaded on different hydrogels under chondrogenic medium at 7 and 14 days in vitro (n = 3). Significant differences symbols: * = *p* < 0.05; ** = *p* < 0.01.

The CCK-8 assay was implemented to quantitatively investigate the proliferation of BMSCs on GelMA/SF composite hydrogels at 1, 3, and 5 days after seeding. As shown in Figure 4A, all hydrogels supported cell growth and showed favorable biocompatibility, as optical density (OD) increased within 5 days in all groups. On day 1, however, there was no statistically significant difference among the groups. However, the pure GelMA hydrogel group showed the highest OD on days 3 and 5, with statistically significant differences among all the hydrogels. In addition, groups with higher SF concentration had lower OD, suggesting decreased cell metabolic activities. Previous studies with similar results suggested that this phenomenon might be related to increased adhesion due to increased GelMA concentration [9,20].

Live/dead staining was conducted to evaluate the adhesion and viability of the BMSCs seeded on the hydrogel (Figure 4B). At 6 h, cells began to attach to the hydrogel surfaces in all groups. GelMA groups had the most cells attached at every time point, while the number of cells attached increased over time in all groups. The cells remained alive, and no significant cytotoxic effect was observed for up to 24 h.

To investigate the biological activity of the four groups of hydrogels on chondrogenic differentiation of BMSCs in vitro, cell-seeded hydrogels were assessed by RT-qPCR (Figure 4C). At 7 days, gene expression levels of *Sox9*, *Acan*, and *Col2a1* in the GelMA/SF-1, GelMA/SF-3, and GelMA/SF-5 groups were significantly higher than that in the GelMA group (*p* < 0.05). Additionally, the gene expression level of *Col1a1* was significantly higher than that in the GelMA group, while that in GelMA/SF-1 had no statistical difference from that in the GelMA group. At 14 days, the target gene expression levels were the highest in the GelMA/SF-5 group. All target gene expression levels in the three composite hydrogel groups were significantly higher than that in GelMA hydrogel (*p* < 0.05).

### 3.4. Macroscopic and Histological Analysis of Osteochondral Defect Repair In Vivo

**Figure 5:**
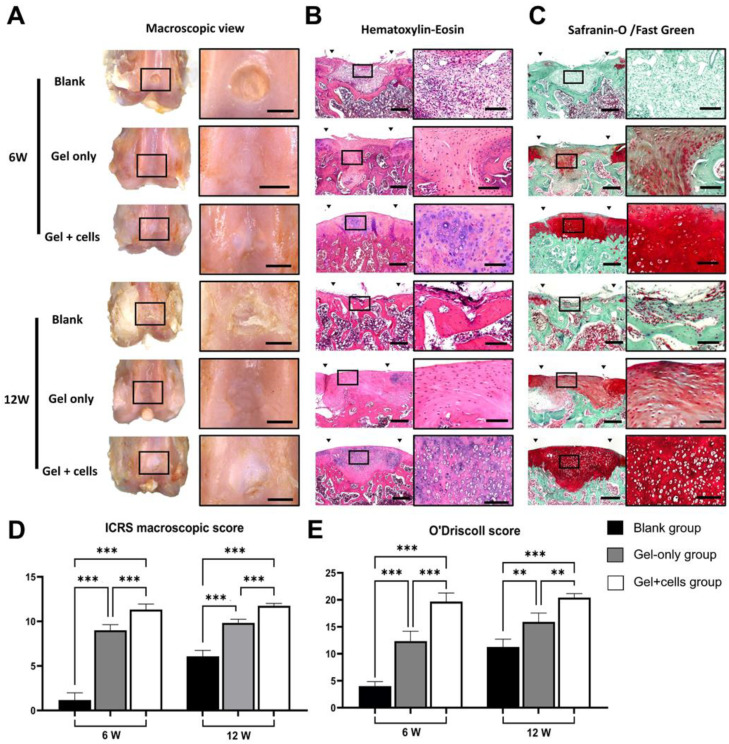
Macroscopic and histological analysis of osteochondral defect repair in vivo. (**A**) Representative images of macroscopic view of samples in each group at 6 and 12 weeks after surgery. (**B**) Representative images of hematoxylin-eosin staining. (**C**) Representative images of safranin-O/fast green staining. (**D**,**E**) ICRS and O’Driscoll scores of each group (n = 6). The Gel + cells groups had the highest scores with statistically significant differences at 6 and 12 weeks. Scale bar = 1 mm in (**A**), 200 μm in (**B**,**C**), and 50 μm in enlargement images in (**B**,**C**); significant differences symbols: ** = *p* < 0.01; *** = *p* < 0.001.

As described in the methods part, 10-week-old male SD rats were randomly divided into three groups (6 knees per group). Different groups were defined as follows: Blank group (defect only, untreated), Gel only group (defect filled with GelMA/SF hydrogel), and Gel + Cells group (defect filled with BMSCs loaded GelMA/SF hydrogel). No death, infections, or rejections of animals were observed until the end time. The macroscopic results at 6 and 12 weeks after surgery are shown in Figure 5A. Harvested samples showed different degrees of repair in osteochondral defect sites. At 6 weeks after surgery, the blank group showed obvious defects, while Gel-only and Gel + cells groups exhibited defects covered by translucent or white-colored repair tissue. At 12 weeks, defects in all three groups were filled. However, blank group samples showed uneven and rough surfaces with fissures and cracks. The repaired tissue in the Gel-only group filled the defect area, but the boundary remained evident. In the Gel + cells group, smooth surface and articular cartilage-like tissue were observed in the defect sites. Regeneration was also evaluated by ICRS macroscopic score, including the degree of defect repair, integration to the border zone, and macroscopic appearance (Figure 5D). Results showed that hydrogel implantation groups, with or without cells, scored significantly higher than blank groups. In contrast, the Gel + cells groups had the highest scores with statistically significant differences at 6 and 12 weeks.

The histological results were evaluated by H-E and Safranin-O/Fast green staining (Figure 5B,C). At 6 weeks, the defect site in the Blank group was mainly filled by fibro-like tissue displaying negative Safranin-O staining. The repaired tissue in the Gel-only group showed partially positive Safranin-O staining, indicating that its cartilage-related content was less than in normal cartilage. The neo-cartilage in the Gel + cells group showed strong staining, which was significantly better than other groups. At 12 weeks, the Blank group showed an uneven defect surface covered by disordered, loose repaired tissue without evident hyaline cartilage regeneration. In the Gel-only group, the repaired tissue showed stronger positive staining at 12 weeks than at 6 weeks, indicating that the regeneration progressed with the accumulated cartilage content. However, the Gel-only group staining was still lighter than the repaired tissue in the Gel + cells group, which showed a smooth surface and no significant difference from surrounding normal cartilage. The regeneration efficacy was also confirmed by the O’Driscoll score evaluation (Figure 5E). The score in the Gel + cells group was significantly superior to those in the other groups at 6 and 12 weeks.

**Figure 6:**
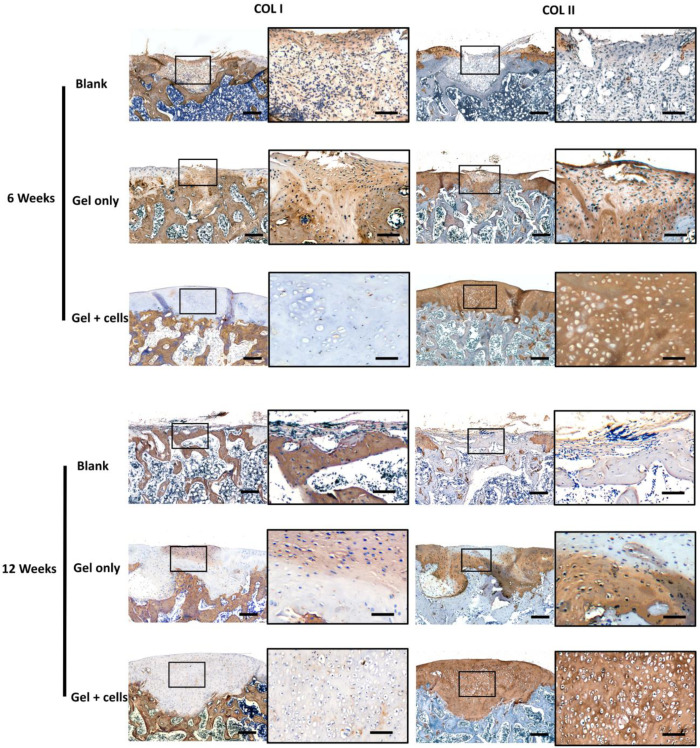
Immunohistochemical staining of collagen type I and II of each sample in different groups. Scale bar = 200 μm and 50 μm in enlargement images. The Gel + cells groups showed strong COLII staining in the cartilage zone and proper COLI staining in the subchondral area, while the Gels-only groups were inferior to native cartilage.

The regeneration was further verified by immunohistochemical staining of collagen type I (COLI) and II (COLII) (Figure 6). The Blank group showed positive COLI staining at 6 weeks and negative COLII staining at both time points, indicating insufficient repair and progressing degradation. The Gels-only group showed consistent results with Safranin-O/Fast green staining, which was inferior to native cartilage. The Gel + cells groups showed strong COLII staining in the cartilage zone and proper COLI staining in the subchondral area.

## 4. Discussion

It is known that articular cartilage has very limited self-healing ability. Early surgical intervention for articular cartilage lesions such as microfracture, usually generates fibro-cartilage tissue [4]. In recent decades, cell-seeded scaffold-based cartilage tissue engineering strategies have been investigated. This study designed a GelMA/SF composite hydrogel as a BMSC-carrier to repair osteochondral defects. The GelMA/SF composite hydrogel showed appropriate mechanical properties, degradation rates, and adhesion ability, making it capable of cartilage regeneration. The in vitro study showed that GelMA/SF hydrogel had favorable biocompatibility, good cell adhesion, and promoted proliferation and chondrogenic differentiation of BMSCs. The hydrogel was implanted into the osteochondral defect sites on the distal femur of rats, with or without BMSCs. The histological analysis showed that the BMSC-seeded hydrogel group yielded satisfactory cartilage and subchondral bone regeneration, while the hydrogel-only group showed partial restoration. Combined, these results suggest that the GelMA/SF composite hydrogel is a feasible strategy for articular cartilage repair.

GelMA, generated from gelatin through methacrylate group incorporation, has been widely studied as an ideal candidate for bioprint technique-based tissue regeneration due to its cost-effectiveness; ease of synthesis; biocompatibility; and improved adhesion, spreading, and proliferation of different cell types [21]. GelMA is a photo-crosslinkable hydrogel gelatinized under ultraviolet (UV) or visible light in the presence of a photo-crosslinker such as LAP (CAS no.85073-19-4) due to the methacryloyl groups. However, articular cartilage is a weight-bearing tissue attached to the joint’s surface, requiring implanted materials with capable hardness. Therefore, the high-load requirements of articular cartilage are difficult to meet by the weak mechanical properties of GelMA, limiting its application in cartilage tissue engineering. To improve this, it would be effective to incorporate other functional constituents. Silk fibroin, the core protein extracted from silkworm silk, has favorable biocompatibility, low immunogenicity, tunable degradation rates, and mechanical properties [22]. It has been widely studied for bone, cartilage, ligament, and tendon tissue engineering applications [23]. GelMA and silk fibroin composite form an IPN with enhanced mechanical properties. IPN hydrogels are composed of at least two physically or chemically crosslinked networks [9]. Compared to single network hydrogels, an IPN demonstrated enhanced mechanical properties, such as improved fracture strength, elongation to break, and compressive properties [24]. This study demonstrates that the composite hydrogel’s compress modulus is tunable with different SF concentrations. The formation of the IPN structure significantly improved the mechanical properties of composite hydrogels, making it possible for further translation and practical application.

Adhesion and integration of implanted materials with native tissue are important in cartilage tissue repair because insufficient adhesion and integration challenge implant stability and the ability of cells to migrate across a mechanically unstable gap [25,26]. Previously reported strategies using sutures, glue pretreatment, or implant surface modification techniques to improve graft–host bonding have shown dissatisfactory results, even impairing the healing [25,26,27]. However, GelMA/SF composite hydrogels exhibit unique tissue adhesion capabilities that facilitate integration; for example, in treating corneal perforations [14]. These characteristics are related to the β-sheet structure formed under different UV exposure conditions because the β-sheet structure provides cohesion between the polypeptide strands, offering mechanically rigid and stable characteristics [14,20]. In this study, the hydrogel filled the defect site without any fixation and sealant, and the adhesion to cartilage was confirmed by macroscopic analysis. The in vivo study showed that the hydrogel stayed in situ and supported the BMSCs’ viability for up to 12 weeks.

BMSCs have been widely studied in cartilage repair as a favorable source of seed cells for tissue engineering. When the cartilage injury involves the subchondral bone layer, the native BMSCs will migrate from the bone marrow cavity to the injury site and participate in repair by differentiating into chondrocytes and producing a cartilage-related extracellular matrix. These BMSCs’ characteristics have also been used in clinical surgical treatment strategies for cartilage injuries, such as microfracture, referred to as bone marrow stimulation techniques. However, this approach, relying solely on local BMSC in cartilage repair, is only applicable to small areas of cartilage injury, and the regeneration produces mostly fibrocartilage rather than hyaline cartilage, with poor long-term efficacy. These findings are consistent with the histological results of the blank group with defects exceeding the critical size in the in vivo experiments. As a result, although the untreated cartilage defect may show a partially restored subchondral bone structure, the articular surface area is often filled with fibrocartilage, resulting in a dissatisfactory repair effect. A prospective randomized controlled clinical study found that patients treated with autologous BMSC transplantation had significantly higher scores at 48 weeks postoperatively than patients treated with microfracture surgery [28]. This indicates that implanted BMSCs play a key role in the repair process. Therefore, we synthesized composite GelMA/SF hydrogel as a cell carrier to provide a favorable chondrogenic differentiation microenvironment for implanted BMSCs. Previous studies have reported that GelMA and SF promoted chondrogenic differentiation of BMSCs [29,30]. In vitro experiments showed that the expressions of chondrogenic-related genes such as Col2a1 and Sox9 were upregulated in BMSCs compared to monolayer culture. The in vivo study showed that the repaired tissue of BMSC-seeded hydrogel groups showed stronger safranin-O and collagen type II staining at both time points compared to the hydrogel-only groups, confirming the chondrogenesis of BMSC-seeded hydrogels. These results suggest that BMSCs encapsulated in hydrogel promote articular cartilage repair.

This study has some limitations. First, we used knee osteochondral defects in rat models because it is more feasible and efficient for preliminary in vivo studies. However, extended studies in large animal models should be performed in the future to confirm its clinical potential. Furthermore, the redistribution and migration of BMSCs within the hydrogel were not fully elucidated and, therefore, need further work. In future experiments, small molecules that promote cartilage differentiation can be added to the hydrogel to achieve a faster and better repair effect.

## 5. Conclusions

In this study, SF was combined with GelMA to fabricate a series of photo-crosslinkable GelMA-SF IPN hydrogels for articular cartilage repair. In vitro studies showed that the formation of the IPN structure did not influence the biocompatibility and cell adhesion of hydrogels. Moreover, these properties could be tailored by changing the concentrations of the components. With higher silk fibroin concentrations (up to 50 mg/mL), the IPN composite hydrogels have demonstrated less porous microstructure, higher mechanical properties, and reduced degradation rate and swelling ratios. The IPN hydrogels promoted the chondrogenesis of BMSCs in vitro as well. An in vivo study was performed to confirm that the GelMA-SF IPN hydrogel could promote cartilage regeneration. The results showed partial regeneration of cartilage in groups treated with hydrogels only but satisfactory cartilage repair in groups of cell-seeded hydrogels, indicating the necessity of additional seeding cells in hydrogel-based cartilage treatment. Therefore, our results suggest that the GelMA/SF IPN hydrogels may be a potential functional material in cartilage repair and regeneration.

## Figures and Tables

**Table 1:** The different formulas for each hydrogel.

Group	GelMA (mg/mL)	Silk Fibroin (mg/mL)	GelMA + SF (mg/mL)
GelMA	60	0	60
GelMA/SF-1	60	10	70
GelMA/SF-3	60	30	90
GelMA/SF-5	60	50	110

**Table 2:** Primer sequences used for chondrogenesis analysis.

Gene	RNA ID	Forward (5′-3′)	Reverse (5′-3′)
Gapdh	NM_017008.4	CTCTCTGCTCCTCCCTGTTC	TACGGCCAAATCCGTTCACA
Sox9	NM_080403.2	TGACTACACCGACCACCAGA	ACTCTGTCACCATTGCTCTTCA
Acan	NM_022190.2	AAGGGCGAGTGGAATGATGT	CGTTTGTAGGTGGTGGCTGTG
Col2a1	XM_006242308.4	GCCAGGATGCCCGAAAATTA	GGCTCCGGGAATACCATCAG
Col1a1	NM_053304.1	CCCTACCCAGCACCTTCAAA	GTGGCCGATGTTTCCAGTCT

## Data Availability

The data supporting this study’s findings are available upon reasonable request from the corresponding author.
